# Melt-Processable Semicrystalline Polyimides Based on 1,4-Bis(3,4-dicarboxyphenoxy)benzene Dianhydride (HQDPA): Synthesis, Crystallization, and Melting Behavior

**DOI:** 10.3390/polym9090420

**Published:** 2017-09-06

**Authors:** Hongfei Zhang, Wei Wang, Guofei Chen, Anjiang Zhang, Xingzhong Fang

**Affiliations:** 1 Key Laboratory of Additive Manufacturing Materials of Zhejiang Province, Ningbo Institute of Material Technology and Engineering, Chinese Academy of Sciences, 1219 Zhongguan West Rd, Zhenhai District, Ningbo 315201, Zhejiang, China; zhanghf@nimte.ac.cn (H.Z.); gfchen@nimte.ac.cn (G.C.); zhanganjiang@nimte.ac.cn (A.Z.); 2 University of Chinese Academy of Sciences, 19 A Yuquan Rd, Shijingshan District, Beijing 100049, China

**Keywords:** semicrystalline polyimide, melt-processable, crystallization kinetics, thermal stability

## Abstract

It is a great challenge to develop semicrystalline polyimides exhibited significant recrystallization ability and fast crystallization kinetics from the melt. A series of semicrystalline polyimides based on 1,4-bis(3,4-dicarboxyphenoxy)benzene dianhydride (HQDPA) and different diamines, including 1,3-bis(4-aminophenoxy)benzene (TPER), 1,4-bis(4-aminophenoxy)benzene (TPEQ), 4,4′-oxydianiline (4,4′-ODA) and 4,4′-bis(4-aminophenoxy)biphenyl (BAPB), end capped with phthalic anhydride were synthesized. Crystallization and melting behaviors were investigated by differential scanning calorimetry (DSC). The polyimide derived from HQDPA/TPER (PI-1) exhibited a glass transition temperature (*T*_g_) at 190 °C and double melting temperatures (*T*_m_s) at 331 °C and 350 °C, and the polyimide derived from HQDPA/TPEQ (PI-2) displayed a *T*_g_ at 214 °C and a *T*_m_ at 388 °C. PI-1 and PI-2 showed significant recrystallization ability from melt and high crystallization rate by isothermal crystallization kinetics study, while polyimides based on 4,4′-ODA and BAPB lost crystallizability once taken to the melt. These polyimides also exhibited excellent thermo-oxidative stability with 5% weight loss temperature higher than 500 °C and good mechanical properties with tensile moduli of 2.0–3.3 GPa, tensile strengths of 85–105 MPa and elongations at break of 5–18%. PI-1 also possessed outstanding melt flowability with less than 300 Pa·s around 370 °C by rheological measurements.

## 1. Introduction

Due to the excellent thermal stability, chemical resistance, and mechanical properties, the polyimides engineering plastic have been widely used in high-temperature application fields such as aerospace, machinery, chemical engineering, electronics, and so on [1,2,3]. However, the rigidity of the backbone structure of polyimides results in most of aromatic polyimides being insoluble and infusible, which makes them extremely difficult to be melt processed. So, the traditional processing method of polyimide engineering plastics is usually by hot-press to get boards and sticks, and then manufactured to different kinds of parts [3]. However, this method is more expensive and time-consuming than the extrusion and injection. Therefore, development of melt-processable thermoplastic polyimides is of prime interest [4,5]. 

Since most commercial thermoplastic polyimides are amorphous, their ultimate working temperatures are limited by glass transition temperatures (*T*_g_), which usually do not exceed 250 °C. However, the introduction of crystallinity could serve as an effective approach to improve the thermal stability, solvent resistance, and mechanical properties of polymers. Hence, development of semicrystalline polyimides has attracted considerable attention for these years [6,7,8,9,10,11,12,13,14,15,16,17,18,19,20,21,22]. The most well-known semicrystalline polyimides include LaRC-CPI developed by NASA [23,24,25,26,27,28], New-TPI developed by Mitsui Toatsu Chemicals [29,30,31,32], R-BAPB type polyimides developed by the Russian Academy of Sciences [33,34,35,36], and some other semicrystalline polyimides developed by the University of Akron [37,38,39,40] and Virginia Polytechnic Institute [41,42,43,44,45]. Although these polyimides exhibit crystallinity, most of them can only crystallize in the presence of solvent and cannot recrystallize once taken to the melt [41]. Only a few polyimides are able to recrystallize from melt, and it was found that the chemical structures of these polyimides tend to have the following characteristics. Firstly, backbone structures always contain ether, carbonyl or biphenyl connecting groups [13,14,23,24,31,32,33,34,35]. Secondly, diamines containing flexible ether groups such as 3,4′-oxydianiline (3,4′-ODA), 1,3-bis(4-aminophenoxy)benzene (TPER), and 4,4′-bis(4-aminophenoxy) biphenyl (BAPB) and their isomers are preferred [8,9,10,11,12,14,29,30,31,32,33,34,35,41,42,43,44,45]. In particular, it was found that the polyimides based on TPER or 3,4′-ODA exhibited excellent melt recrystallization ability and high crystallization rates [14,41,42]. Thirdly, the molecular weights are controlled ranged from 10 k to 30 k [41,42,43,44,45].

However, some problems make these polyimides difficult to satisfy the actual requirements of melt-processable semicrystalline polyimides. Firstly, the recrystallization ability of polyimides decreases evidently after being melt for a few times or melt at high temperatures, like LaRC-CPI and R-BAPB type polyimides [23,34]. Secondly, some polyimides exhibit quite slow melt crystallization rates, the products from injection molding are usually amorphous without long time heat treatments, like New-TPI [29,30,31,32]. Thirdly, the melting temperatures of most semicrystalline polyimides are always too high to be melting processed. Most of the previous studies focused on polyimides based on rigid dianhydride monomers such as 3,3′,4,4′-biphenyltetracarboxylic dianhydride (BPDA), 3,3′,4,4′-benzophenonetetracarboxylic dianhydride (BTDA), pyromellitic dianhydride (PMDA), etc., the melting points of these polymers are always too high for melt processing [29,30,31,32,41,42,43,44]. For example, melting points of semicrystalline polyimides based on BPDA usually exceed 390 °C [14,42,44]. So, it is important to develop novel melt-processable semicrystalline polyimides to address these issues from both a fundamental and practical standpoint. 

As is known, 1,4-bis(3,4-dicarboxyphenoxy)benzene dianhydride (HQDPA) based polyimides have been mostly used as amorphous materials [46,47]. An earlier study [48] seemed to indicate that this kind of polyimide might exhibit crystallizability. Two flexible ether linkages of HQDPA would improve the mobility of molecular chains, which can not only increase the melt crystallization rates, but also can decrease the melting points and melt viscosities. So it makes HQDPA based polyimides more attractive for melt processing. In this paper, a series of semicrystalline polyimides derived from HQDPA and aromatic diamines with different numbers of benzene rings, different isomeric substituted positions were synthesized. The crystallization behaviors, thermal stability, melt processability, mechanical properties of polyimides were systematic investigated. Some structures exhibited excellent recrystallization ability from melt and melt-processability. These semicrystalline polyimides might become a new generation of high performance engineering plastics in the future. The purpose of our work is to reveal the relationship between crystallinity of polyimides and structures of diamines, and eventually to develop novel semicrystalline polyimides with appropriate melting temperatures and excellent recrystallization ability from melt.

## 2. Materials and Methods

### 2.1. Materials

1,3-Bis(4-aminophenoxy)benzene (TPER), 1,4-bis(4-aminophenoxy)benzene (TPEQ), and 4,4′-bis(4-aminophenoxy)biphenyl (BAPB) were supplied by Changzhou Sunshine Chemical Corp. 4,4′-Oxydianiline (4,4′-ODA) was purchased from Shandong Wanda Chemical Company. The 1,4-bis(3,4-dicarboxyphenoxy)benzene dianhydride (HQDPA) was obtained from Hipolyking Corp. and dried at 150 °C prior to use. Phthalic anhydride (PA), isoquinoline and *m*-cresol were obtained from Aladdin and used as received. All other reagents for the study were commercially obtained and used as received without further purification.

### 2.2. Synthesis of Polymers

The semicrystalline polyimides based on HQDPA were synthesized via one-step polycondensation in *m*-cresol. A typical synthesis procedure for the polyimide derived from HQDPA/TPEQ (PI-2) was carried out as follows:

A 250-mL, three-necked flask fitted with a nitrogen inlet tube and a condenser with a Dean-Stark trap was used as the reaction vessel. HQDPA (7.7244 g, 19.2 mmol), TPEQ (5.8466 g, 20.0 mmol) and PA (0.2370 g, 1.6 mmol) were firstly added to the reaction vessel. *m*-Cresol was added to achieve a 10% solid content, and then a few drops of isoquinoline were added successively as the catalyst. The reaction mixture was reacted at 200 °C for 8 h under nitrogen to afford the polyimide. After cooling down, ethanol was added to the reaction mixture. The precipitated polyimide was collected by filtration, followed by thorough washing with ethanol and dried in a vacuum oven at 200 °C for 2 h. The inherent viscosity of PI-2 was 0.58 dL/g in *m*-cresol at a concentration of 0.5 g/dL at 30 °C. The other polyimides were also synthesized by the same procedure as described above.

### 2.3. Polymer Film Preparation

The polyimide films were prepared by heating the polyimide powder at about 30 °C higher than melting temperature for 10 min under 9.8 MPa load followed by slowly cooling to room temperature. 

### 2.4. Measurements

The inherent viscosity (*η*_inh_) of the polyimide was determined using an Ubbelohde viscometer with capillary diameter of 1.07 mm at a concentration of 0.5 g/dL in *m*-cresol at 30 °C. The Fourier transform infrared spectra (FT-IR) were obtained from a Thermo Scientific Nicolet 6700 FT-IR spectrometer (Thermo Fisher Scientific, Grand Island, NY, USA) and the sample was prepared with KBr pellets. Thermogravimetric analysis (TGA) studies were carried out on a TG/DTA (Mettler Toledo, Zurich, Switzerland). The powder samples were tested at a heating rate of 10 °C·min^−1^ under either a nitrogen or air atmosphere. The tensile measurements were measured using an Instron model 5567 (Instron, Boston, MA, USA), the film specimens (50 mm long, 10 mm wide, and about 30 μm thick) were tested at a strain rate of 5 mm·min^−1^ at room temperature. The differential scanning calorimetry (DSC) measurements were conducted on a DSC (Mettler Toledo, Zurich, Switzerland), 6–8 mg of sample under a nitrogen purge, at heating rates of 10 °C·min^−1^ unless otherwise specified. The DSC curves shown in this paper have been normalized to 1 mg sample mass. Temperature and heat flow were calibrated using indium and zinc standards. Wide-angle X-ray diffraction (WAXD) measurement of the powder was carried out via Bruker D8 Advance Davinci X-ray Powder Diffraction (Bruker, Rheinstetten, Germany) with Cu Kα radiation. The data were collected in a fixed time mode with a step size of 0.02° (2θ) and run in the range 2θ = 10–50°, operated at 40 KV and 40 mA. Dynamic mechanical thermal analysis (DMA) was performed on a dynamic mechanical thermal analyzer DMA Q800 (TA Instruments, Eden Prairie, MN, USA) in a tensile mode at a heating rate of 3 °C·min^−1^ and at a frequency of 1 Hz in air. The rheological properties of polyimides were performed on a rotational rheometer Physica MCR 301 (Anton Paar, Graz, Austria) in an oscillation model at a heating rate of 3 °C·min^−1^ and at a frequency of 1 Hz in air. Polarized optical microscopic (POM) was performed on Olympus BX51 optical microscope (Olympus, Tokyo, Japan) equipped with a hot stage, a video camera. The measurements were performed on thin films sandwiched between two microscope cover slips.

## 3. Results and Discussion

### 3.1. Polymer Synthesis

**Scheme 1:**
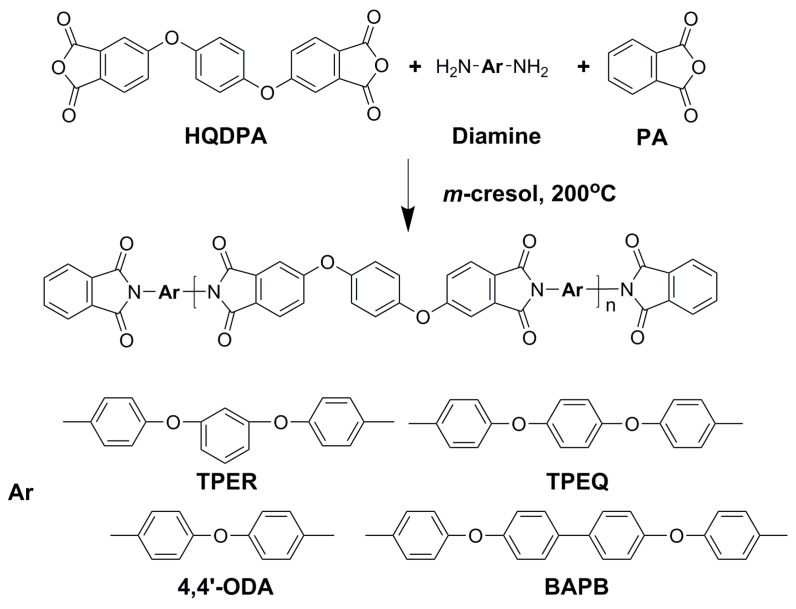
Synthesis of polyimides derived from 1,4-bis(3,4-dicarboxyphenoxy)benzene dianhydride (HQDPA).

Polyimides were synthesized from HQDPA with four different diamines (TPER, TPEQ, 4,4′-ODA, and BAPB) and coded as PI-1, PI-2, PI-3, and PI-4, respectively (Scheme 1) [49]. Stoichiometric imbalances were used to control the molecular weight of polyimides, and the PA was used as an end capper for terminating polymer chains. The theoretical calculated molecular weights of PI-(1–4) are about *M*_n_ = 15,000 Da, which is suitable for melt processing and high enough to demonstrate excellent thermal resistance and mechanical properties. The monomer composition and inherent viscosity (*η*_inh_) of polyimides are summarized in Table 1. The *η*_inh_ of the polyimides are in the range of 0.43–0.58 dL/g. The Fourier transform infrared spectra confirmed that all the polyimides were fully imidized as shown in Figure S1. The peaks at around 1720 and 1380 cm^−1^ correspond to the C=O symmetric stretch and C–N stretch of imide, respectively. 

### 3.2. Thermal Stability

**Figure 1:**
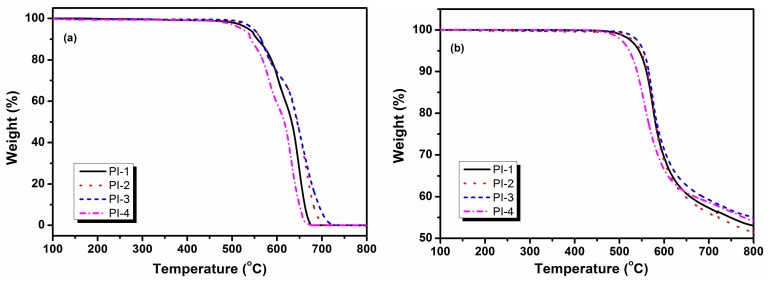
TGA curves of polyimides: (**a**) in Air; (**b**) in N_2_.

TGA was utilized to evaluate the thermal stability of polyimides and TGA curves of polyimides in different atmospheres are shown in Figure 1. The temperatures of 5% weight loss for all the polyimides under nitrogen and air are summarized in Table 1. All the polyimides exhibited excellent thermal stability in air and nitrogen. The temperatures of 5% weight loss of polyimides range from 525–553 °C in nitrogen and 522–547 °C in air, respectively. The residual weight retention at 800 °C in nitrogen for all the samples are more than 50%. 

### 3.3. Wide-Angle X-ray Diffraction

**Figure 2:**
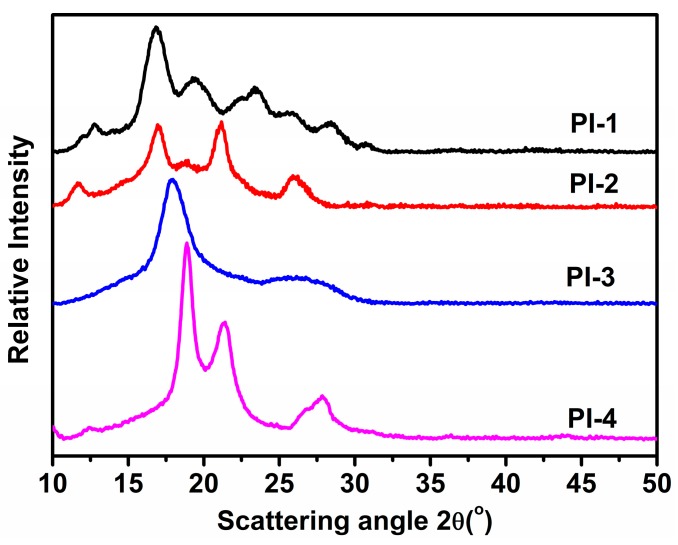
Wide-angle X-ray diffraction (WAXD) patterns of polyimide powders.

The crystal structures of the polyimides were studied by wide-angle X-ray diffraction (WAXD). Figure 2 shows WAXD patterns for as-made polyimide powder specimens. Obvious diffraction peaks above the broad amorphous halo suggested that all the polyimides had a semicrystalline character. However, the diffraction peak positions changed gradually with the variation of diamines. 

### 3.4. Melting Behavior

DSC experiments were conducted to study the melting behaviors and recrystallization ability of polyimides. 

#### 3.4.1. Initial Melting Behavior

**Figure 3:**
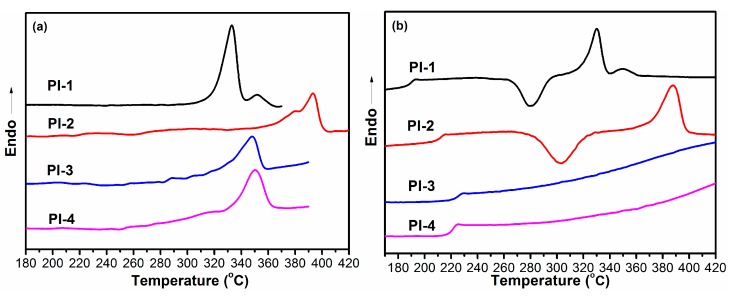
DSC curves for HQDPA polyimides: (**a**) first heating; (**b**) second heating.

The first DSC heating scans for the as-made polyimide powders are shown in Figure 3a. All the samples exhibited prominent melting endotherms with peak temperatures ranging from 349 °C to 393 °C, which suggested that all of the polyimide powders were crystalline, while the specific heat transitions corresponding to *T*_g_ were not observed. The crystallization properties of polyimides are summarized in Table 1. 

#### 3.4.2. Second Melting Behavior

Since the thermal history has influence on the first DSC heating scan, samples were first heated to 30 °C above the melting temperature and held for 3 min to eliminate the thermal history. And then the samples were quenched to ambient temperature at a rate of 200 °C·min^−1^ and reheated at a rate of 10 °C·min^−1^. The second heating scans for the quenched polyimides are shown in Figure 3b. It is worth emphasizing that neither PI-1 nor PI-2 could be quenched into a pure amorphous state, implying that both of them exhibit excellent crystallization ability from melt, while the melting endotherms of PI-3 and PI-4 cannot be observed any longer. The heat fusion of PI-1 and PI-2 was estimated to be 52 J/g and 35 J/g, respectively. From the Figure 3b, it can also be seen that PI-1 exhibited a prominent *T*_g_ at 190 °C, followed by a cold crystallization exotherm (*T*_c_) at 285 °C, and double melting endotherms (*T*_m_) at 331 °C and 350 °C, while the melting temperature of the polyimide base on BPDA/TPER is 395 °C [41]. The lower melting temperature of PI-1 can be attributed to the flexible dianhydride (HQDPA) containing double ether linkages, which could significantly improve the mobility of the molecular chain. PI-2 displayed a *T*_m_ of 388 °C, which was close to that of New-TPI (*T*_m_ = 385 °C) [31]. It can be seen that *T*_m_ of PI-2 from *para*-linked diamine (TPEQ) are higher than that of PI-1 from *meta*-linked diamine (TPER). Comparing with PI-2, the melting temperature of PI-1 is lower than 360 °C, which makes it more convenient for melt processing. It was very easy to quench the polyimide based on 4,4′-oxydiphthalic anhydride (ODPA)/TPEQ into a purely amorphous state [45], however, the quenched PI-1 and PI-2 still could exhibit sharp melting endotherms, implying the excellent crystallization ability. The recrystallization ability of PI-3 and PI-4 are not very good, they can only exhibit crystallizability during the first heating scan. 

In summary, it was found that PI-3 and PI-4 could not recrystallize once taken to the melt, while PI-1 and PI-2 exhibited recrystallization ability from melt, which made them attractive candidates for high-performance melt-processable engineering plastics. Therefore, more detailed characterization like the repeated melting behavior, non-isothermal crystallization behavior, isothermal crystallization kinetics, and dynamic thermomechanical analysis of PI-1 and PI-2 were carried out.

#### 3.4.3. Repeated Melting Behavior

**Figure 4:**
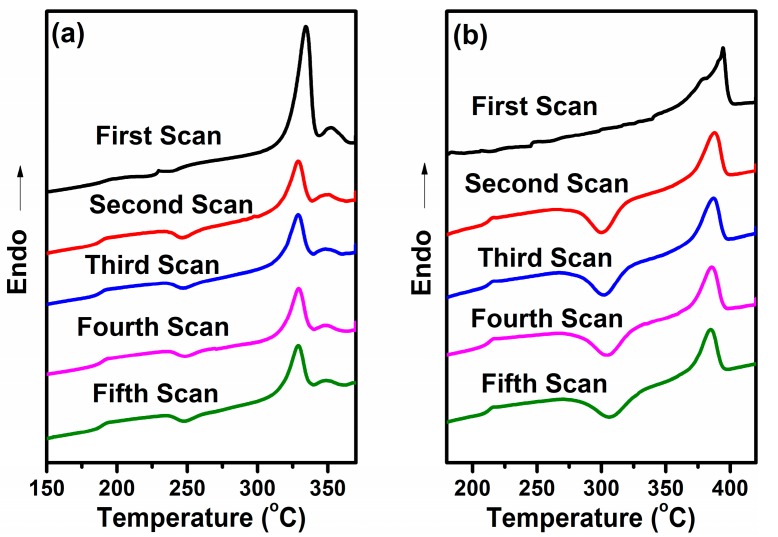
Repeatedly multiple DSC heating scans of (**a**) PI-1; (**b**) PI-2.

The recrystallization ability of PI-1 and PI-2 were then carefully studied by repeatedly DSC heating scans after being melted for 3 min several times. As shown in Figure 4a, the as-made PI-1 is crystalline and displays prominent double melting endotherms without a clear *T*_g_. However, the *T*_g_ of PI-1 around 180–200 °C was observed during the subsequent four rescans. A very weak recrystallization exotherm with peak temperatures of 250 °C and double melting endotherms with peak temperatures of 330 °C and 350 °C were also observed. The similar phenomenon was also observed for PI-2 in Figure 4b. The as-made PI-2 has been proven to be crystalline, however, a *T*_g_ in the range from 210 °C to 220 °C and recrystallization exotherms with peak temperatures of 300 °C to 310 °C were observed at the subsequent four rescans, and the melting endotherm was found to be 390 °C. The recrystallization exotherms of PI-2 were more obvious than that of PI-1, which implied the crystallization rate of PI-2 was much slower than PI-1. Both of PI-1 and PI-2 haven’t exhibited any obvious changes in DSC curves after repeatedly melt, indicating they exhibited excellent recrystallization ability from melt. 

According to the melting behavior study above, we can see that PI-1 exhibited lower melting temperatures and excellent recrystallization ability from the melt, it can become a potential melt-processable semicrystalline polyimides.

### 3.5. Nonisothermal Crystallization Behavior

**Figure 5:**
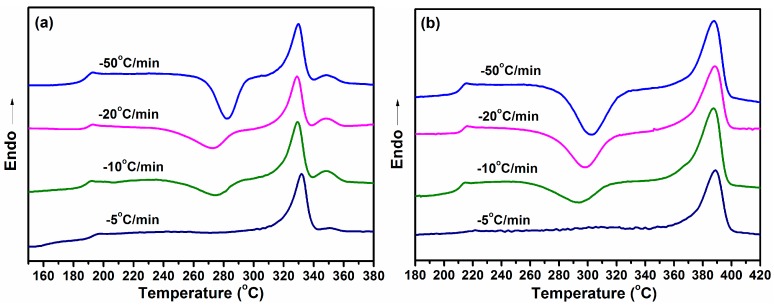
DSC scans for samples subjected to various cooling rates from the melt: (**a**) PI-1; (**b**) PI-2.

The effect of different cooling rates on the subsequent melting behavior was also investigated. Figure 5a,b show DSC heating scans of PI-1 and PI-2 at 10 °C·min^−1^ following various cooling rates from the melt, respectively. PI-1 and PI-2 could crystallize during cooling at a cooling rate 5 °C·min^−1^, as is evident from the lack of a clear glass transition and absence of a crystallization exotherm in the heating scans. With the increasing of cooling rates, the glass transition and the crystallization exotherm become more obvious. The crystallization rate of PI-1 is also found to be faster than PI-2, as is evident by the crystallization exotherm of PI-1 is less obvious than PI-2 after the same cooling treatment. 

### 3.6. Isothermal Crystallization Behavior

**Figure 6:**
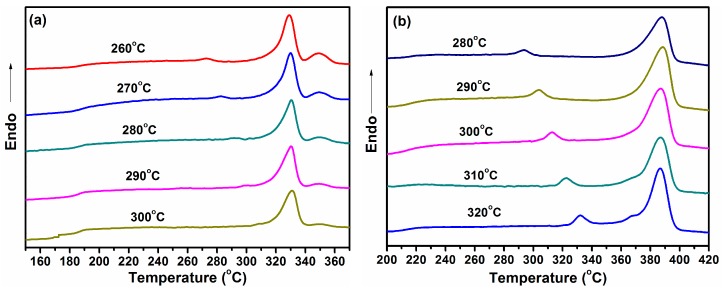
DSC scans for samples isothermally crystallized at various temperatures: (**a**) PI-1; (**b**) PI-2.

Isothermal experiments were conducted to obtain more detailed information about the crystallization kinetics and the crystallization mechanism. The polyimides were firstly heated at 20 °C·min^−1^ to the melting temperature and held for 3 min, then were cooled at 200 °C·min^−1^ to various crystallization temperatures and held for 30 min, and were then cooled to ambient temperature at a rate of 200 °C·min^−1^. The samples were then reheated at a rate of 10 °C·min^−1^. Figure 6a shows the DSC scans of PI-1 after crystallizing at temperatures ranging from 260 °C to 300 °C. As can be seen, the melting behavior of PI-1 includes triple melting endotherms. The first endotherm (assigned as Peak I) appears at about 15 °C higher than the previous *T*_c_. The second endotherm (Peak II) and third endotherm (Peak III) present at about 330 and 350 °C, respectively. With the increasing of *T*_c_, the peak II shifts to higher temperatures, while the peak III gradually became indistinct. 

Figure 6b shows the heating scans of PI-2 after being crystallized at temperatures ranging from 280 °C to 320 °C, and the PI-2 presents double melting endotherms. The first endotherm (Peak I) also appeared at about 15 °C higher than the previous *T*_c_. The second endotherm (Peak II) showed at about 390 °C, which is independent of the previous crystallization temperatures. 

The Peak I of PI-1 and PI-2 could be attributed to the “annealing peak”, which results from much poorer crystals growing between the larger crystals [50]. In order to explore the reason for multiple melting behavior of PI-1, WAXD, and DSC experiments were conducted according to the literature [27]. PI-1 was subjected to isotherm treatment at 340 °C between its two melting peaks (the temperature of Peak II was about 330 °C and the temperature of Peak III was about 350 °C). The DSC curves of PI-1 after different isothermal treatment is shown in Figure S2. After the isothermal treatment, the endothermic peaks at high temperature gradually became stronger, while the endothermic peak at low temperature gradually decreased. The reason for that may be due to the high temperature heat treatment, the crystal lamellae with lower melting temperature and less perfect crystal melted during the isothermal heat treatment, and the molecular chain rearrangement to form more perfect crystal, so the enthalpy of endothermic peaks at high temperature increases. 

WAXD was also conducted after PI-1 was isothermally treated by DSC. The WAXD patterns of PI-1 after isothermal treatment are shown in Figure S3. The XRD patterns of PI-1 after isothermally treatment at 340 °C are similar to that isothermally treatment at 260 °C. The positions of diffraction peaks had no significant changes. There were no new diffraction peaks, indicating that no new crystal structures were formed. According to the above study, the reason for the multiple melting behavior may be the difference in crystal size.

#### 3.6.1. Equilibrium Melting Temperatures

It was necessary to determine the equilibrium melting temperatures (*T*_m_^o^) of PI-1 and PI-2. The *T*_m_^o^ is defined as the melting temperature of infinitely thick crystals, and it can be obtained by the Hoffman-Weeks equation [40]. Using the equation, *T*_m_^o^ can be determined by extrapolating the plot of *T*_m_ as a function *T*_c_ to the *T*_m_ = *T*_c_ line. As is shown in Figure S4, *T*_m_^o^ was determined to be 356 °C and 390 °C for PI-1 and PI-2, respectively. Based on this result, samples for measuring isothermal crystallization kinetics were heated above *T*_m_^o^ to ensure complete melting.

#### 3.6.2. Isothermal Crystallization Kinetics

Avrami analysis is the most widely used means for describing the overall bulk isothermal crystallization of polymers. It can provide important information about the crystallization mechanism and kinetics parameters. The Avrami equation [51] is generally written as:

*X*_c_ (*t*) = 1 − exp(−*Kt^n^*) (1)

where *X*_c_ (*t*) is the relative degree of crystallinity at time *t*; the parameter *K* is a composite rate constant involving both nucleation and growth rate parameters; and *n* is the Avrami exponent, which indicates the nucleation mechanism and growth dimensions. The Equation (1) could also express in the form of Equation (2).

log [−ln(1 − *X*_c_ (*t*))] = log *K* + *n* log *t* (2)

The values of *n* and *K* can be determined by the slope and intercept of the initial portion. Although, the value of *K* can be used to estimate the bulk crystallization rate, the units of *K* depend on the value of *n*. So the values of *K*^1/*n*^ are always used to evaluate the crystallization rate. Another important parameter is the crystallization half-time, *t*_1/2_, which is defined as the time at which 50% crystallization is completed and is determined from the measured kinetic parameters.

*t*_1/2_ = (ln 2/*k*)^1/*n*^ (3)

Usually, the rate of crystallization, *τ*_1/2_, is described as the reciprocal of *t*_1/2_. 

*τ*_1/2_ = 1/*t*_1/2_ (4)

**Figure 7:**
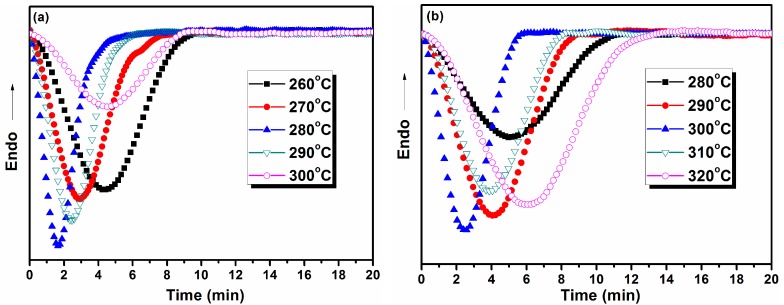
Heat flow versus time for the isothermal crystallization of polyimides at various crystallization temperatures. (**a**) PI-1; (**b**) PI-2.

The heat flow versus time and the relative crystallinity *X*_c_(*t*) as a function of time for isothermal crystallization at various temperatures for PI-1 and PI-2 are shown in Figure 7 and Figure S5, respectively. The plot of log [−ln (1 − *X*_c_ (*t*))] versus log *t* is shown in Figure S6. The value of *K*, *n*, *t*_1/2_, and other kinetic parameters for PI-1 and PI-2 are listed in Table 2. For PI-1, the values of *n* vary from 2.3 to 2.6 with the *T*_c_ increasing from 260 °C to 300 °C. For the PI-2, the values of *n* vary from 2.3 to 2.9 with the *T*_c_ increasing from 280 °C to 320 °C. The results suggested that the primary crystallization processes should correspond to a three-dimensional spherulitic growth or a two-dimensional circular diffusion-controlled growth.

**Figure 8:**
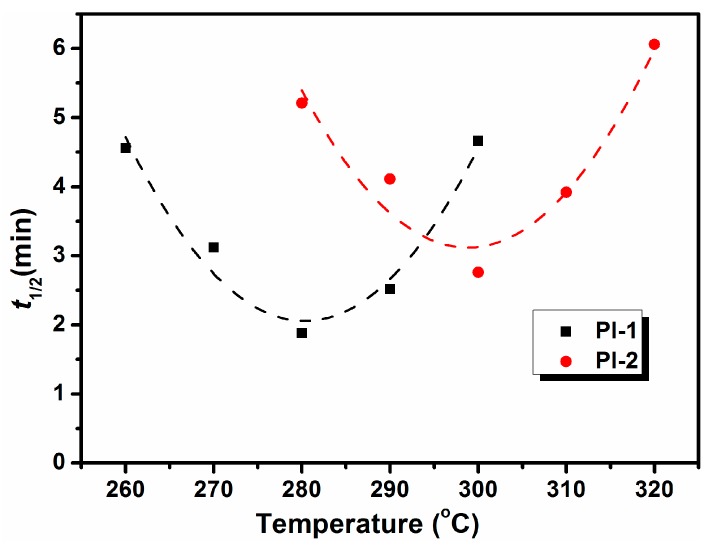
The crystallization half-time (*t*_1/2_) vs. crystallization temperature plot.

As evident in Figure 7, the overall bulk crystallization of PI-1 is very temperature sensitive in the narrow 40 °C range from 260–300 °C. The value of *K*^1/*n*^ increases first with the increasing of *T*_c_ and then decreases. The *τ*_1/2_ also showed to have the same tendency of changes with the increasing of *T*_c_. PI-2 had the similar tendency of changes as PI-1 with the increasing of *T*_c_. The crystallization rate reached a maximum (*T*_max_) at 280 °C and 300 °C for PI-1 and PI-2, respectively. Figure 8 demonstrates the relationship between *t*_1/2_ and crystallization temperatures of PI-1 and PI-2. There is a minimum of *t*_1/2_ for each polymer, corresponding to the fastest crystallization rate. The fastest crystallization rate of PI-1 can be observed at *T*_c_ = 280 °C, and for PI-2 it is at *T*_c_ = 300 °C.

The reasons for the change of crystallization rate could be because the crystallization rate is determined by the nucleation forming rate at temperature above *T*_max_, which decreases with the increasing of *T*_c_; while the crystallization rate is determined by the nucleation growth rate at temperature blow *T*_max_, which increases with the increasing of *T*_c_ [52].

#### 3.6.3. Comparison with Other Semicrystalline Polymers

In order to compare the crystallization kinetic parameters with other semicrystalline polymers, the results from other reports and this work are summarized in Table 3. The values of *K*^1/n^ of PI-1 and PI-2 are higher than that of PEEK and New-TPI, indicating that both of them possess relatively higher crystallization rate than these polymers. 

### 3.7. Crystalline Morphology

**Figure 9:**
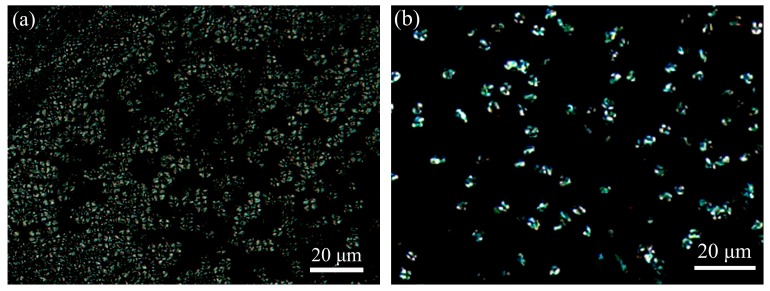
Polarized optical microscopic (POM) observations of the crystal morphologies: (**a**) PI-1 crystallized at 280 °C for 5 min; (**b**) PI-2 crystallized at 300 °C for 5 min.

Polarized optical microscopic (POM) observations of PI-1 and PI-2 have been conducted to investigate the crystal morphology and the results are shown in Figure 9. The Maltese cross could be observed for both of PI-1 and PI-2, which proved the growth of spherulite. The result was consistent with the Avrami analysis, which suggested that the primary crystallization processes should correspond to a three-dimensional spherulitic growth or a two-dimensional circular diffusion-controlled growth. The spherulite size of PI-1, after crystallized at 280 °C for 5 min, was larger than that of PI-2 crystallized at 300 °C for 5 min, which proved that the crystallization growth rate of PI-1 was higher than PI-2.

### 3.8. Melt Processability

**Figure 10:**
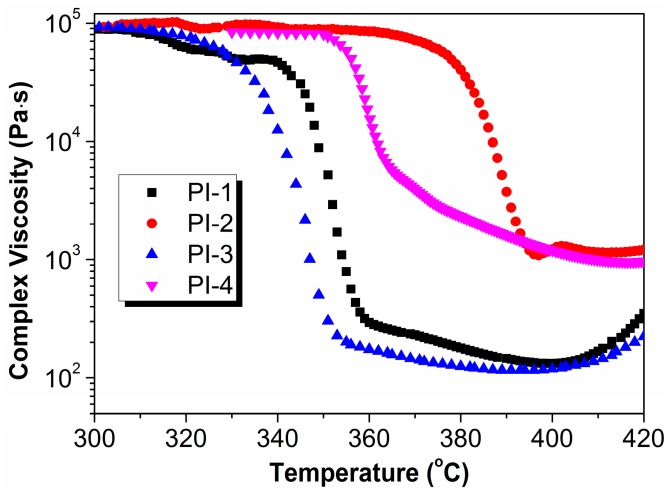
The melt viscosities as a function of temperature for polyimides.

In order to evaluate the melt processability of polyimides, rheological measurements of polyimides were conducted from 300 °C to 450 °C. The melt viscosities as a function of temperature are plotted in Figure 10. With the increase of the temperature, the complex viscosity of polyimides first decreased and then increased. All the polyimides maintain high initial complex viscosity around 8 × 10^4^ Pa·s to 1 × 10^5^ Pa·s until the temperature approaches their melting temperatures, and then the viscosity decreased dramatically. PI-2 and PI-4 showed higher complex viscosity around 1200 Pa·s around 400 °C because of the more rigid structure. While the complex viscosity of PI-1 and PI-3 were less than 300 Pa·s around 370 °C, indicating that they were more suitable for melt processing. When temperature reached above 400 °C, the melt viscosities of PI-1 and PI-3 increased continuously, this may be due to chain extension or crosslinking reactions in the melt [14,42]. All of the polyimides are soluble in the *m*-cresol with solid content of 0.5 wt % before the rheological test. However, it was found that PI-1 and PI-3 became insoluble in *m*-cresol after the rheological test. It could be inferred that the molecular chain had cross-linked in some degree. 

Since air atmosphere was used during the rheological test, the color of polymers changed from white to dark brown at the end of the rheological test. The sample may undergo cross-linking processes by oxidation in the air atmosphere, leading to a graphite like charred residue as the temperature increases. The cross-linked fraction becomes insoluble. Thermal cleavage of the ether units to yield oligomers with 3-hydroxyphthalimide end groups and oligomers with phenyl end groups may be hypothesized to occur in the thermal oxidation [54].

**Figure 11:**
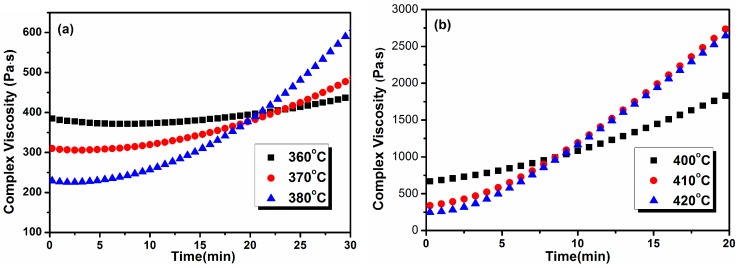
The melt viscosities as a function of time for polyimides: (**a**) PI-1; (**b**) PI-2.

Isothermal melt viscosity studies were also carried out on powder samples. Figure 11a,b show the isothermal complex viscosity of PI-1 and PI-2 at different temperatures, respectively. It was observed that their initial viscosity was very sensitive to melt temperature. The initial complex viscosity of PI-1 was about 400 Pa·s at 360 °C, and it decreased to 240 Pa·s at 380 °C. The complex viscosity did not show any obvious increase for times up to 30 min at 360 °C. While the melt viscosity after 30 min at 380 °C was almost 2.5 times higher than that after 5 min. The viscosity of the PI-2 was too high and it increased very fast when temperatures were higher than 410 °C. It was found that the melt viscosity of the semicrystalline polyimide BPDA/TPER was significantly increased during isothermal rheological test. The main reason for that is due to crosslinking/chain branching [43]. It was also found that PI-1 and PI-2 became insoluble in *m*-cresol after the rheological test. It may due to the crosslinking reactions in the melt.

The results by rheological studies indicate that PI-1 has appropriate melt viscosity and excellent melt stability, and it is more suitable for melt processing. The minimum melt complex viscosity of PI-1 was lower than the R-BAPB type of polyimide, indicating that PI-1 could be a potential candidate for high performance thermoplastic resin matrix for composites [35].

### 3.9. Mechanical Properties

**Figure 12:**
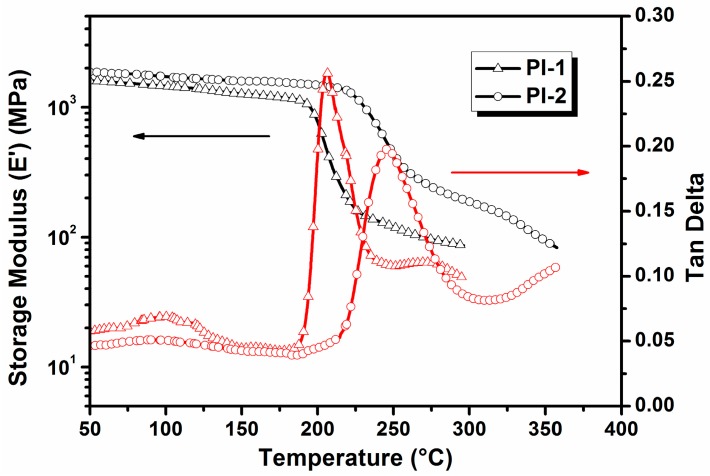
Dynamic mechanical thermal analysis (DMA) curves for polyimides.

Table 4 summarizes the mechanical properties of the polyimide films. All the films exhibit excellent mechanical properties with tensile modulus of 2.0–3.3 GPa, tensile strength of 85–105 MPa and elongation at break of 5–18%. In order to test the mechanical properties of PI-1 and PI-2 at high temperatures, their viscoelastic behaviors were analyzed by dynamic mechanical thermal analysis (DMA) measurements. The curves of storage modulus and tan δ versus temperature are shown in Figure 12. The peak temperatures of tan *δ* were used to represent *T*_g_s of polyimide films, and the *T*_g_s of PI-1 and PI-2 films are 205 °C and 245 °C, respectively. The *T*_g_ values obtained by DMA are higher than that measured by DSC. This could be attributed to the different responses of the samples to the two characterization methods. PI-1 and PI-2 maintain an initial storage modulus around 2.0 GPa and the storage modulus drops gradually when temperature reached their *T*_g_s and maintain a relatively stable plateau around 150 MPa until melting temperature. This is different from amorphous polymers, whose storage modulus decreased sharply above *T*_g_. The storage modulus curves indicated that the PI-1 and PI-2 possess outstanding mechanical performance under high temperature and could maintain their structural stiffness up to 300 and 360 °C, respectively. This indicated that both of them can be used as high-temperature semicrystalline polymers.

## 4. Conclusions

The synthesis and characterization of a series of semicrystalline polyimides based on 1,4-bis(3,4-dicarboxyphenoxy) benzene dianhydride have been reported. All polyimides exhibited excellent thermal stability and mechanical properties. In particular, PI-1 and PI-2 displayed significant recrystallization ability and fast crystallization kinetics from the melt. The PI-1 exhibited *T*_g_ of 190 °C and double *T*_m_ at 331 °C and 350 °C, while the PI-2 displayed *T*_g_ and *T*_m_ at 214 °C, and 388 °C, respectively. *T*_m_^o^s were found to be 356 °C and 390 °C for PI-1 and PI-2 by the Hoffman-Weeks equation, respectively. The value of *K*^1/n^ of PI-1 and PI-2 obtained from Avrami analysis are higher than that of PEEK and New-TPI, indicating the high crystallization rate from melt. Furthermore, PI-1 and PI-2 also exhibited excellent melt-processability and mechanical performance under high temperature, which made them attractive candidates for high-performance melt-processable engineering plastics. 

## Figures and Tables

**Table 1:** Inherent viscosities, thermal properties, and crystallization properties of polyimides.

Sample	Diamine	*η*_inh_^a^ (dL/g)	*T*_5%_ (°C) ^b^		*R*_800_^c^ (%)	*T*_g_^d^ (°C)	*T*_m_^e^ (°C)	*T*_m_ * ^f^ (°C)
			N_2_	Air				
PI-1	TPER	0.43	540	536	52.6	190	333,351	331,350
PI-2	TPEQ	0.58	548	545	51.5	214	393	388
PI-3	4,4′-ODA	0.46	553	547	56.3	223	349	-
PI-4	BAPB	0.56	525	522	54.1	223	354	-

^a^ The inherent viscosities measured at a concentration of 0.5 g/dL in *m*-cresol at 30 °C; ^b^ Temperature of 5% weight loss were recorded by thermogravimetric analysis (TGA) at a heating rate of 10 °C·min^−1^ in nitrogen or air; ^c^ Residual weight retention at 800 °C in nitrogen; ^d^ Measured in the second differential scanning calorimetry (DSC) scans at a heating rate of 10 °C·min^−1^ in nitrogen; ^e^ Melting point measured by first DSC heating scan at a heating rate of 10 °C·min^−1^; ^f^ Melting point measured by second DSC heating scan at a heating rate of 10 °C·min^−1^ after melting for 3 min.

**Table 2:** Kinetic parameters for PI-1 and PI-2 obtained from isothermal crystallization.

Sample	*T*_c_^a^ (°C)	*n*	*K* (min^−*n*^)	*K*^1/*n*^	*t*_1/2_ (min)	*τ*_1/2_ (min^−1^)
PI-1	260	2.5	0.0149	0.19	4.56	0.22
	270	2.4	0.0464	0.27	3.12	0.32
	280	2.3	0.1649	0.45	1.88	0.53
	290	2.5	0.0694	0.34	2.52	0.40
	300	2.6	0.0123	0.19	4.66	0.21
PI-2	280	2.5	0.0115	0.17	5.21	0.19
	290	2.7	0.0163	0.21	4.11	0.24
	300	2.3	0.0658	0.31	2.76	0.36
	310	2.8	0.0147	0.22	3.92	0.26
	320	2.9	0.0039	0.15	6.06	0.17

^a^ Crystallization temperature.

**Table 3:** Bulk crystallization rates for some semicrystalline polymers.

Polymer	Melt Temperature (°C)	Undercooling, Δ*T* (°C)	*K*^1/*n*^	Ref.
PEEK	410	80	0.06	[53]
New-TPI	410	121	0.04	[31]
BPDA/TPER	430	55	0.61	[43]
PI-1	370	76	0.45	This work
PI-2	420	90	0.31	This work

**Table 4:** Mechanical properties of polyimides.

Sample	Tensile Strength (MPa)	Tensile Modulus (GPa)	Elongation at Break (%)
PI-1	97	3.1	5
PI-2	105	3.3	5
PI-3	103	2.5	18
PI-4	85	2.0	10

## Supplementary Materials

The following are available online at www.mdpi.com/2073-4360/9/9/420/s1. Figure S1: FTIR spectra of polyimides. Figure S2: DSC curves of PI-1 after different isothermal treatment. Figure S3: WAXD patterns of PI-1 after different isothermal treatment. Figure S4: Hoffman-Weeks plot for PI-1 and PI-2. Figure S5: Normalized crystalline content as a function of time at various crystallization temperatures. (a) PI-1, (b) PI-2. Figure S6: Avrami plot of log [−ln (1-*X*_c_ (*t*))] versus log *t* at various crystallization temperatures. (a) PI-1, (b) PI-2.
