# Supplementary material for: Melt-Processable Semicrystalline Polyimides Based on 1,4-Bis(3,4-dicarboxyphenoxy)benzene Dianhydride (HQDPA): Synthesis, Crystallization, and Melting Behavior
Source: Polymers (Basel). 2017 Sep 6;9(9):420. doi: 10.3390/polym9090420 (PMC6418867; doi:10.3390/polym9090420)
Supplement: Supplementary file 1 [file polymers-09-00420-s001.pdf]

Melt-Processable Semicrystalline Polyimides Based on 1,4-Bis(3,4-dicarboxyphenoxy)benzene Dianhydride (HQDPA): Synthesis, Crystallization and Melting Behavior

Hongfei Zhang[1,2], Wei Wang[1], Guofei Chen[1], Anjiang Zhang[1], Xingzhong Fang[1] *

[1] Key Laboratory of Additive Manufacturing Materials of Zhejiang Province, Ningbo Institute of Material Technology and Engineering, Chinese Academy of Sciences, 1219 Zhongguan West Rd, Zhenhai District, Ningbo, Zhejiang, 315201, China.; fxzhong@nimte.ac.cn

[2] University of Chinese Academy of Sciences, 19 A Yuquan Rd, Shijingshan District, Beijing, 100049, China.; zhanghf@nimte.ac.cn

* Correspondence: fxzhong@nimte.ac.cn; Tel.: +86-574-86685185

Supplementary Materials:

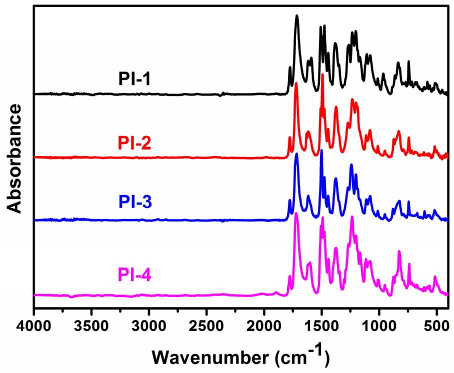

Figure S1. FTIR spectra of polyimides

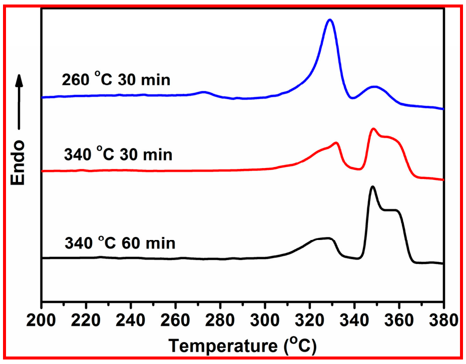

Figure S2. DSC curves of PI-1 after different isothermal treatment.

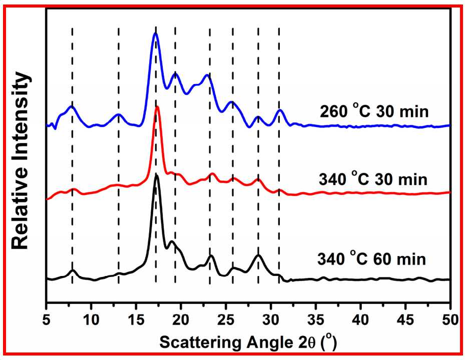

Figure S3. WAXD patterns of PI-1 after different isothermal treatment

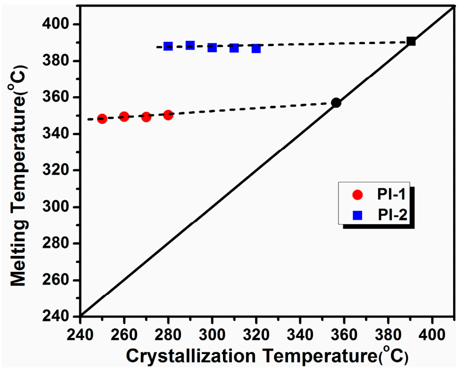

Figure S4. Hoffman-Weeks plot for PI-1 and PI-2.

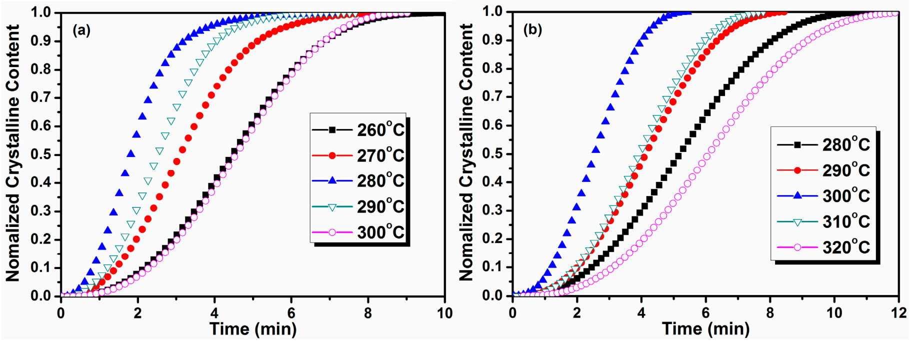

Figure S5. Normalized crystalline content as a function of time at various crystallization temperatures. (a) PI-1, (b) PI-2.

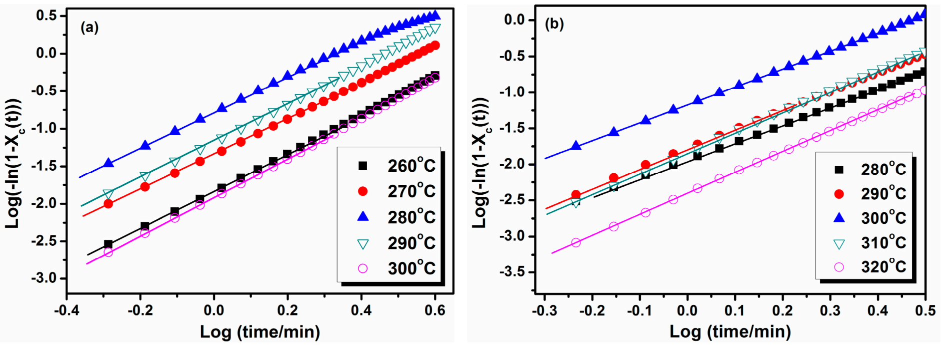

Figure S6. Avrami plot of log [-ln (1-X_c (t))] versus log t at various crystallization temperatures. (a) PI-1, (b) PI-2.
